# Hematopoietic Stem Cell-Derived Adipocytes Modulate Adipose Tissue Cellularity, Leptin Production and Insulin Responsiveness in Female Mice

**DOI:** 10.3389/fendo.2022.844877

**Published:** 2022-06-03

**Authors:** Kathleen M. Gavin, Timothy M. Sullivan, Joanne K. Maltzahn, Matthew R. Jackman, Andrew E. Libby, Paul S. MacLean, Wendy M. Kohrt, Susan M. Majka, Dwight J. Klemm

**Affiliations:** ^1^ Geriatric Research, Education and Clinical Center, Rocky Mountain Regional Veterans Administration (VA) Medical Center, Aurora, CO, United States; ^2^ Division of Geriatric Medicine, Department of Medicine, University of Colorado Anschutz Medical Campus, Aurora, CO, United States; ^3^ Cardiovascular Pulmonary Research Laboratory, University of Colorado Anschutz Medical Campus, Aurora, CO, United States; ^4^ Division of Endocrinology, Department of Medicine, University of Colorado Anschutz Medical Campus, Aurora, CO, United States; ^5^ Division of Pulmonary, Critical Care and Sleep Medicine, Department of Biomedical Research, National Jewish Health, Denver, CO, United States; ^6^ Charles C. Gates Center for Regenerative Medicine and Stem Cell Biology, University of Colorado Anschutz Medical Campus, Aurora, CO, United States

**Keywords:** adipocyte, ablation, leptin, insulin resistance, physical activity, cellularity

## Abstract

A subpopulation of adipocytes in the major adipose depots of mice is produced from hematopoietic stem cells rather than mesenchymal progenitors that are the source of conventional white and brown/beige adipocytes. To analyze the impact of hematopoietic stem cell-derived adipocytes (HSCDAs) in the adipose niche we transplanted HSCs in which expression of a diphtheria toxin gene was under the control of the adipocyte-specific adiponectin gene promoter into irradiated wild type recipients. Thus, only adipocytes produced from HSC would be ablated while conventional white and brown adipocytes produced from mesenchymal progenitor cells would be spared. Wild type mice transplanted with HSCs from mice containing a reporter gene, but not the diphtheria toxin gene, regulated by the adiponectin gene promoter served as controls. In mice in which HSCDA production was suppressed, adipocyte size declined while adipose depot weights were unchanged and the number of conventional adipocyte progenitors significantly increased. We also measured a paradoxical increase in circulating leptin levels while physical activity was significantly decreased in the HSCDA depleted mice. Finally, insulin sensitivity was significantly reduced in HSCDA depleted mice. In contrast, loss of HSCDA production had no effect on body weight, components of energy balance, or levels of several circulating adipokines and tissue-resident inflammatory cells. These data indicate that ablation of this low-abundance subpopulation of adipocytes is associated with changes in circulating leptin levels and leptin-regulated endpoints associated with adipose tissue function. How they do so remains a mystery, but our results highlight the need for additional studies to explore the role of HSCDAs in other physiologic contexts such as obesity, metabolic dysfunction or loss of sex hormone production.

## Introduction

Adipose tissue was once believed to be a homogenous and metabolically dormant tissue. It is now recognized, not only for its central role in the storage and controlled release of dietary energy, but also its participation in the regulation of energy metabolism, satiety, physical activity and inflammation throughout the body (1). Adipose tissue also exhibits substantial cellular heterogeneity between different body locations, and within the same depot (2–8). This depot-specific heterogeneity appears to be due primarily to the progression of mesenchymal stem cells along different developmental pathways to produce the functionally and spatially distinct adipocyte populations (8).

While it was originally assumed that all adipocytes were derived from the mesenchymal lineage there is now substantial evidence that a portion of adipocytes are generated from the hematopoietic lineage in mice (9–11) and humans (12). These cells were originally discovered in wild-type mice that received bone marrow transplants from donor mice constitutively expressing green fluorescent protein (GFP) (9, 10). The existence of these bone marrow-derived adipocytes was subsequently confirmed in non-myeloablative models (13, 14). In mice their abundance slowly increases for at least 11 months (14). Bone marrow-derived adipocytes have also been detected in human bone marrow recipients where their abundance appears to increase for more than 4 years (12). Bone marrow-derived adipocytes are more abundant in female than male mice, and in visceral rather than subcutaneous adipose depots (9, 10). Preliminary gene expression analysis indicated that these cells may produce high levels of inflammatory cytokines, but little or no leptin. Thus, we hypothesized that bone marrow derived adipocytes may negatively influence satiety and energy balance through their reduced leptin secretion and contribute to local and systemic inflammation through elevated cytokine production. Using competitive bone marrow transplants and non-myeloablative lineage tracking models we demonstrated that these adipocytes were derived from hematopoietic stem cells *via* the myeloid lineage (10, 12–15) and now refer to them as Hematopoietic Stem Cell-Derived Adipocytes (HSCDAs).

A substantial portion of what is known about adipose tissue function is a result of genetically manipulated mouse models in which most, if not all adipocytes including white and brown/beige adipocytes were ablated (16–19). Similar strategies have been used to selectively ablate brown/beige adipocytes while maintaining the production of white adipocytes, confirming the role of brown/beige fat cells in thermogenic energy expenditure (20, 21). Likewise, significant insights have been obtained in novel single nuclei and single cell RNA-seq studies of adipose tissue subpopulations (22, 23). These experiments have highlighted the fact that adipocytes and their progenitors comprise multiple subpopulations with distinct pattern of gene expression in individual depots. However, there are no current models to address specific functional phenotypic characteristics of white adipocyte subpopulations. Genetic markers for these subpopulations are not restricted to the adipocyte lineage, and their use in ablation models would target terminal mesenchymal lineages beyond adipocytes.

Here, we leveraged the production of HSCDAs from hematopoietic progenitors to create mice in which HSCDA production was genetically diminished, while the production of conventional white and brown/beige adipocytes was unimpeded. This was achieved through adoptive transfer of donor BM hematopoietic stem cells (HSCs) from mice in which expression of a diphtheria toxin subunit A gene was controlled by the adipocyte-restricted adiponectin gene promoter. This model allowed us to ask how large of an impact do HSCDAs have under normal conditions on whole body metabolism and inflammation, and adipose tissue cellularity. Blockade of HSCDA production had no effect on energy intake or energy expenditure, nor did it alter common adipose tissue inflammatory endpoints as we had previously predicted. Instead, loss of HSCDA production was accompanied by changes in adipose tissue cellularity that indicate that suppression of HSCDA production is accompanied by their replacement with conventional adipocytes. In addition, suppression of HSCDA production was accompanied by elevated circulating leptin levels, decreased physical activity and insulin resistance. Thus, although HSCDAs are a relatively minor subpopulation of adipose tissue, inhibiting their production elicited significant changes in endpoints commonly associated with hyperleptinemia. The results suggest that individual adipocyte subpopulations derived from distinct developmental pathways may each influence a subset of structural endpoints and physiologic processes as they contribute to overall adipose tissue/depot function.

## Results

### Targeted Depletion of HSCDAs Impacts the Conventional Adipocyte Niche

**Figure 1:**
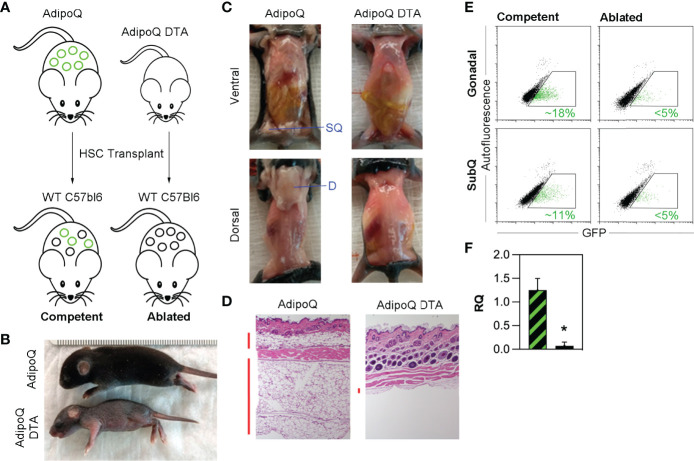
Triple transgenic model for specific depletion of HSCDAs. **(A)** AdipoQ donor mice were generated by crossing mice in which the adiponectin gene promoter (AdipoQ) drove cre recombinase expression with mice bearing a floxed tdTomato and EGFP reporter gene at the ROSA locus (ROSA^mTmG/+^). AdipoQ DTA donor mice also contained the AdipoQ cre and floxed EGFP genes, plus a floxed diphtheria toxin subunit a (DTA) transgene (ROSA^mTmG/DTA^). These mice were used as donors for HSC transplant in female, wild-type C57Bl/6J recipient mice to generate HSCDA Competent and Ablated mice for experiments. Conventional adipocytes are shown as black circles and HSCDAs as green circles **(B)** Donor AdipoQ DTA pups were substantially smaller than AdipoQ donor mice of the same age. **(C)** AdipoQ DTA donor mice lacked dorsal and inguinal subQ adipose tissue, which were present in the AdipoQ mice. The position of each fat depot is indicated by blue label and line. **(D)** Brightfield microscope images (10x) of hematoxylin and eosin stained skin cross sections show normal levels of subdermal adipocytes in AdipoQ mice, but almost none in AdipoQ DTA mice. Vertical red bars to the left of each image highlight the adipocyte layers. **(E)** Flow cytometry of free-floating adipocytes from the gonadal and subcutaneous adipose tissue from each HSC recipient cohort revealed the presence of GFP^POS^ HSCDAs in the Competent mice (green events in the gated region, average GFP^POS^ HSCDAs indicated in green font), which were significantly reduced in the Ablated cohort. **(F)** Quantitative RT-PCR of GFP gene expression in gonadal adipose tissue samples from Competent (black/green crosshatch bar) and Ablated (solid black bar) mice. GFP signals were corrected for β2-microglobulin RNA levels. (n=3 per group, * indicates p≤0.05).

Selective depletion of HSCDAs was achieved by engineering bone marrow HSC donor mice in which adipocyte-specific expression of both a diphtheria toxin subunit A (ROSA^DTA^) gene and a mTomato/mGFP reporter gene (ROSA^mTmG^) were controlled by the adipocyte-specific adiponectin gene promoter driving Cre recombinase (AdipoQcre) **(** **Figure 1A** **)**. These transplant donor mice, harboring AdipoQcre, ROSA^mTmG^ and ROSA^DTA^ transgenes were designated “AdipoQ DTA”. Littermates that lacked ROSA^DTA^ but contained the AdipoQcre and ROSA^mTmG^ transgenes served as control donors and were designated “AdipoQ”. This combination was necessary rather than Cre-negative control mice because Cre recombinase was required for GFP expression and verification of successful HSC transplant. AdipoQ and AdipoQ DTA mice served as bone marrow HSC donors for wild-type recipients allowing us to explicitly monitor the presence and function solely of HSCDAs.

The AdipoQ DTA donor pups were significantly smaller than litter- and age-matched AdipoQ donors **(** **Figure 1B** **)**, and completely lacked all adipose depots including the subcutaneous inguinal (ventral) fat around the hind limbs and interscapular (dorsal) fat between the shoulder blades, which was present in normal quantity in AdipoQ pups **(** **Figure 1C** **)**. Additionally, microscopic evaluation of skin sections from AdipoQ DTA mice revealed a complete lack of subcutaneous adipocytes, which were present in AdipoQ skin sections **(** **Figure 1D** **)**. Thus, targeted ablation of adipocytes in AdipoQ DTA mice is efficient and complete.

HSCs were isolated by flow cytometry **(** **Supplementary Figure 1** **)** from whole bone marrow recovered from the femurs and tibias 28-35 day old female littermate AdipoQ and AdipoQ DTA donors and transplanted into separate cohorts of lethally irradiated 12-week old wild-type female recipient mice and allowed to engraft for approximately 4 weeks. Mice receiving HSCs from AdipoQ DTA donors were predicted to produce only conventional adipocytes while HSCDA production would be blocked or reduced. In mice receiving transplants with bone marrow HSC from AdipoQ donors, production of both conventional adipocytes and HSCDAs were predicted to be normal. Flow cytometry 20 weeks post-transplant **(** **Supplementary Figure 2** confirmed AdipoQcre-driven GFP expression in the adipocytes of AdipoQ recipients (~16% in gonadal adipocytes and ~11% in subcutaneous adipocytes) while GFP-expressing adipocytes were greatly diminished in mice transplanted with AdipoQ DTA marrow (<5% in both depots) **(** **Figure 1E** **)**. HSCDA depletion in the Ablated mice was verified by quantitative RT-PCR for the EGFP reporter gene, which was significantly lower in Ablated mice than in the Competent cohort **(** **Figure 1F** **)**. The GFP^POS^ cells detected by flow cytometry and quantitative RT-PCR correlated with the presence of GFP-expressing cells containing large lipid droplets observed by fluorescence microscopy or imaging flow cytometry **(** **Supplementary Figures 3A, B**, respectively**)** in the Competent, but not the Ablated mice. Mice that received HSCs from AdipoQ donors were designated “Competent”, and recipients of HSCs from AdipoQ DTA donors were designated “Ablated” in all figures.

### Blockade of HSCDA Production Increases Conventional Adipocyte Progenitor Abundance

**Figure 2:**
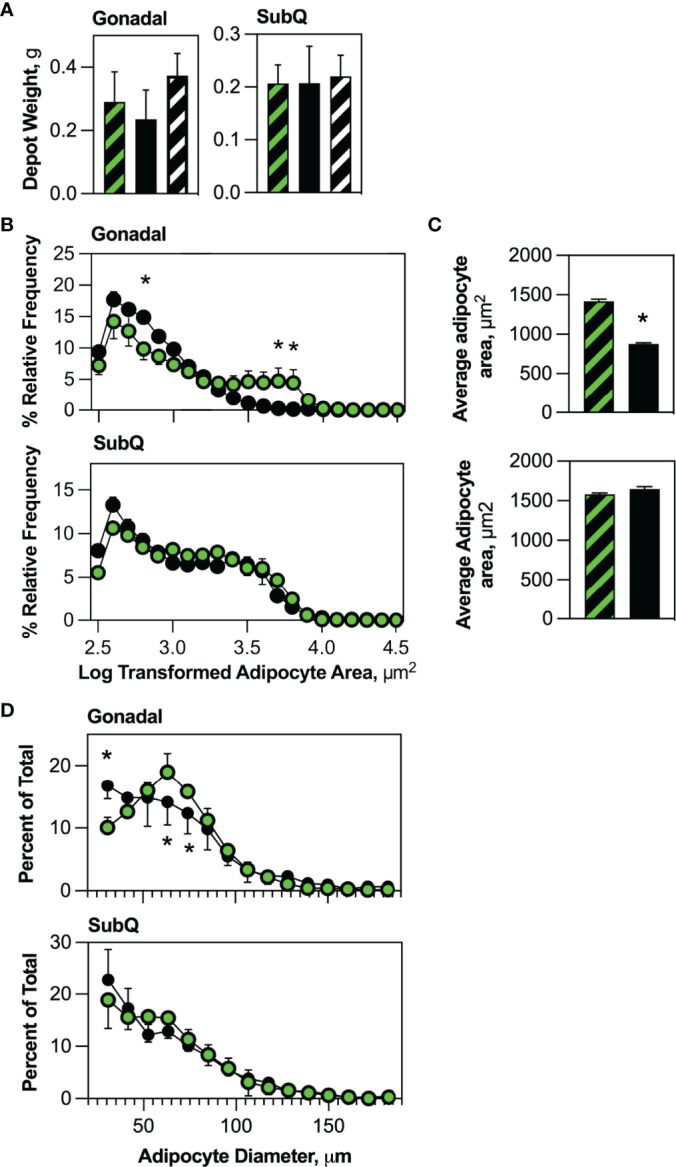
HSCDA depletion did not affect adipose depot weights, but significantly reduced adipocyte size. **(A)** The weight of gonadal and subcutaneous (SubQ) fat pads in transplant-naïve, Competent (black/green crosshatch bars) and Ablated (solid black bars) mice were measured at the end of the experimental regimen. (n=8 per cohort) **(B)** Gonadal and subcutaneous (SubQ) fat was removed from Competent (black/green circles) and Ablated (black circles) mice, paraformaldehyde fixed paraffin embedded tissue sections were prepared and stained with H&E. Adipocyte size was quantified with 10x phase contrast images captured and analyzed with the AdipoSoft plugin in FIJI software. The Log of adipocyte area versus frequency distributions are displayed. (n=8 per cohort, * indicates p≤0.05) **(C)** Adipocyte area was quantified and the average for the entire Competent (black/green circles) and Ablated (black circles**)** is shown. (n=8 per cohort, * indicates p≤0.05) **(D)** Adipocytes from gonadal and subcutaneous (SubQ) adipose depots from Competent (black/green circles) and Ablated (black circles) mice were isolated from tissue by collagenase digestion into single cell suspensions. Dilutions of the adipocytes were immediately counted with a Cellometer Vision imaging cytometer, and the diameter of individual adipocytes measured under brightfield conditions was plotted. (n=8 per cohort, * indicates p≤0.05).

We anticipated blockade of new HSCDA production might reduce adipose tissue mass. Interestingly, we found that the mass of gonadal and subcutaneous adipose depots also did not differ between transplant-naive, Competent or Ablated cohorts **(** **Figure 2A** **)**. Thus, we explored potential changes in adipocyte size and/or abundance. Morphometric analysis of fixed adipose tissue sections from gonadal and subcutaneous depots (representative images are shown in **Supplementary Figure 4**) demonstrated an increase in the abundance of small adipocytes and a decrease in larger adipocytes in the gonadal depot from the Ablated compared to the Competent cohort **(** **Figure 2B**), but not in the subcutaneous fat. This was even more evident when the data was averaged for the entire adipocyte populations **(** **Figure 2C** **)**. Likewise, analysis of free-floating adipocytes from collagenase-digested adipose tissue revealed significantly more small adipocytes ranging from 30 to 40 μm but significantly fewer adipocytes between 60 and 80 μm **(** **Figure 2D** **)** in gonadal adipose tissue of ablated mice, but no significant differences in adipocyte size in subcutaneous fat between cohorts. The increase in small adipocytes and concomitant decline in larger adipocytes in Ablated mice, while adipose depot weights remained the same suggested that production of new conventional fat cells was normalizing adipose tissue mass due to loss of HSCDAs.

**Figure 3:**
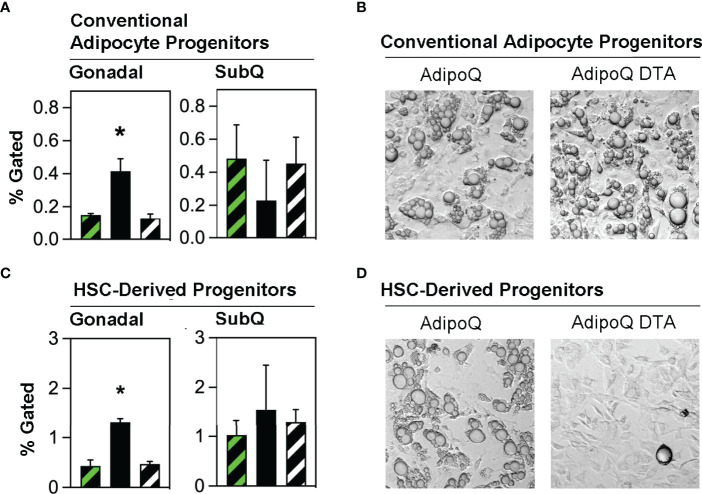
Conventional adipocyte progenitors and HSCDA progenitors are more abundant in the gonadal fat of Ablated mice. **(A)** Gonadal and subcutaneous (SubQ) fat was recovered from transplant-naïve (black/white crosshatch bar), Competent (black/green crosshatch bar) and Ablated (solid black bar) mice at the end of the experiment at 18 weeks. The adipose tissue underwent collagenase digestion and stromal vascular cells were separated and stained with conventional adipocyte and HSCDA lineage marker antibodies and subjected to flow cytometric analysis (gating strategy shown in **Supplementary Figure 4** **).** The proportion of conventional adipocyte progenitors (Lin^NEG^/CD29^POS^/PDGFRα^POS^ cells) are shown here. (n=3 per cohort, * indicates p≤0.05) **(B)** Putative conventional adipocyte progenitors from the gonadal fat of Competent and Ablated mice were purified by flow cytometry sorting and plated on tissue culture plates. The cells from both cohorts spontaneously underwent adipogenic conversion over a period of 7-14 days. Representative 10x brightfield microscope images are shown. **(C)** The abundance of HSCDA progenitors was measured in the gonadal and SubQ fat of transplant-naïve, (black/white crosshatch bar), Competent (black/green crosshatch bar) and Ablated (solid black bar) mice as described in 3A, except that fluorescent antibodies to CD45, CD11b, CD29, PDGFRα and Sca1 were used instead of those described above. (n=3 per cohort, * indicates p≤0.05) **(D)** Putative HSCDA progenitors were isolated by flow cytometry sorting, plated in 3-dimensional fibrin clots and recovered after 5 days. The cells were then re-plated on tissue culture plates and treated with conventional pro-adipogenic culture media to induce adipogenesis for a period of 14 days. Representative 10x brightfield microscope images show robust adipogenic conversion of cells from Competent, but not Ablated mice.

Studies from several laboratories (8, 24) and our group (13, 15) have generally defined conventional adipocyte progenitors as adipose tissue stromal cells lacking hematopoietic lineage markers (defined as Lin^NEG^) while co-expressing mesenchymal markers including CD29, Sca-1 and PDGFRα. We quantified conventional adipocyte progenitors (Lin^NEG^/CD29^POS^/PDGFRα^POS^) cells in the stroma of gonadal and subcutaneous adipose tissue from transplant-naïve, Competent and Ablated mice **(** **Supplementary Figure 5** **)**. These cells were significantly more abundant in the gonadal fat of Ablated than in Competent or transplant naive mice **(** **Figure 3A** **)**. Flow cytometry sorted conventional adipocyte progenitors from Competent and Ablated mice were both capable of spontaneous adipogenesis *in vitro* **(** **Figure 3B** **)**.

We recently reported that HSCDAs are produced from hematopoietic stem cells through intermediate progenitor cells simultaneously expressing both myeloid markers (CD45 and CD11b) and mesenchymal progenitor markers (CD29, Sca-1 and PDGFRα) (15). Adipose stromal cells bearing these markers were also isolated from transplant-naïve, Competent and Ablated mice and found to be significantly more abundant in the gonadal fat of the Ablated mice **(** **Figure 3C** **)**, with no difference noted in subcutaneous fat. When these cells were cultured in fibrin clots to induce their conversion to HSCDAs (13, 15), lipid-engorged adipocytes were only produced from HSCDA progenitors from Competent but not Ablated mice **(** **Figure 3D** **)**, further confirming the ablation model.

### Blockade of HSCDA Depletion Had No Effect on Body Weight or Body Composition

**Figure 4:**
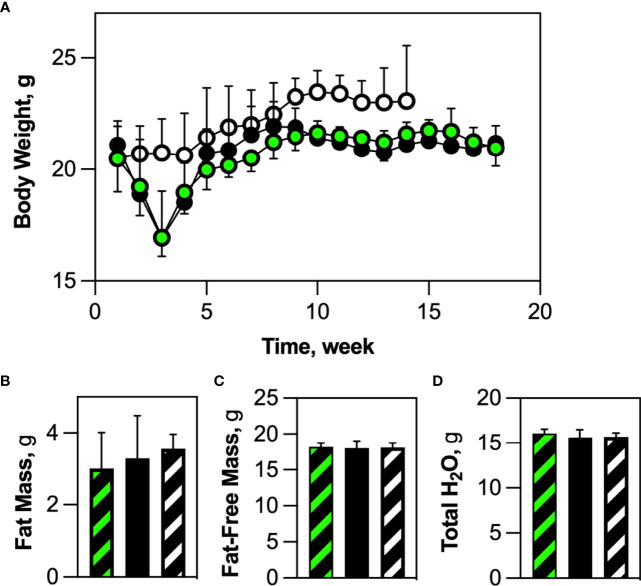
HSCDA depletion did not elicit changes in body weight or composition. **(A)** Wild-type mice were transplanted with HSCs from either AdipoQ or AdipoQ DTA donor mice to generate Competent (black/green circles) or Ablated (black circles) cohorts, respectively. Transplant-naive control mice are indicated by black/white circles. After transplant, mice in all cohorts were weighed weekly. (n=10 per cohort) **(B–D)** 18 weeks (2 weeks recovery + 16 weeks experimental) after transplantation, transplant-naïve, (white/black crosshatch bar), Competent (black/green crosshatch bar) and Ablated (solid black bar) mice were subjected to quantitative magnetic resonance analysis to measure body composition. No changes in percent fat mass, fat-free mass or total water content were observed. (n=8 per cohort).

We originally surmised that ablation of HSCDAs would cause a modest reduction in body weight and adiposity. We found that both Competent and Ablated mice lost body weight for the first 2-3 weeks post-transplant compared to transplant-naïve control mice **(** **Figure 4A** **)**. However, these cohorts regained body weight over the next 5-6 weeks, after which their weights plateaued equally. Likewise, no significant body composition differences were noted between cohorts for adiposity (% Fat, **Figure 4B**), fat-free mass **(** **Figure 4C** **)** or body water content **(** **Figure 4D** **)**. There was also no sign of excess lipid deposition in liver or skeletal muscle **(** **Supplementary Figures 6A, B**, respectively). As noted earlier, HSCDA ablation was associated with decreased adipocyte size, but no change in adipose depot weight/volume. These results suggest that blockade of HSCDA production elicits the generation of new conventional adipocytes, perhaps as a mechanism to normalize adiposity and adipose tissue cellularity.

### Adipokine Production and Inflammatory Cell Abundance in Competent and Ablated Cohorts

**Figure 5:**
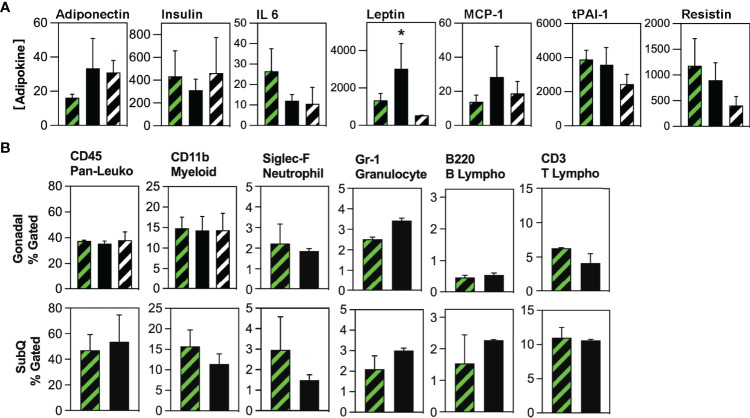
Evaluation of circulating adipokine and adipose tissue-resident immune cell populations in Competent and Ablated mice. **(A)** Circulating adipokine levels were measured in the serum of transplant-naïve (black/white crosshatch bars), Competent (black/green crosshatch bars) and Ablated (solid black bars) mice with Luminex Adipokine Assay reagents, except adiponectin which was measured separately with Luminex Adiponectin Assay reagents. Adiponectin levels are reported in µg/ml, while all other cytokine levels are reported in pg/ml. (n=3 per cohort, * indicates p≤0.05) **(B)** Adipose tissue-resident immune cell populations indicated above each plot were quantified by flow cytometry in the gonadal and subcutaneous (SubQ) fat from transplant-naïve (black/white crosshatch bars)Competent (black/green crosshatch bars) and Ablated (solid black bars) mice. (n=3 per cohort, * indicates p≤0.05).

Global gene expression analysis previously indicated that HSCDAs might possess a detrimental phenotype characterized by elevated transcript levels of inflammatory cytokines and an absence of leptin (9, 10). We translated these findings to our *in vivo* model and analyzed the production of adipokines using a Luminex assay **(** **Figure 5A** **)**. Circulating leptin levels were significantly higher (>3-fold) in Ablated animals. Interleukin-6 (IL-6) expression was substantially reduced and adiponectin was elevated in the Ablated mice although these differences did not achieve statistical significance when the analysis (by ANOVA) included the transplant-naïve cohort. When the transplant-naïve cohort was excluded from the analysis, and data analyzed by unpaired t test, the differences between Competent and Ablated cohorts approached but were shy of p≤0.05. No significant differences were observed between cohorts for serum insulin, macrophage chemoattractant protein-1 (MCP-1), tissue plasminogen activator inhibitor-1 (tPAI-1) or resistin. Tumor necrosis factor-α (TNF-α) was undetectable in all samples.

Because both cohorts received myeloablative conditioning in preparation for HSC transplant, we compared cytokine production between non-irradiated mice and the Competent cohort. Circulating levels of insulin, IL-6 and MCP-1 did not differ in these cohorts **(** **Figure 5A** **)**. Leptin, tPAI-1 and resistin were all significantly lower in the non-irradiated mice, while adiponectin was significantly higher in this cohort. TNF-α was again not detected. Thus, some inflammatory adipokines (tPAI-1 and resistin), but not all (IL-6 and MCP-1) were elevated in myeloablated mice.

Next, we measured the abundance of tissue-resident immune cell populations in the Competent and Ablated cohorts by flow cytometry. No differences were observed in CD45^POS^ (pan-hematopoietic), CD11b^POS^ (myeloid), B220^POS^ (B lymphocyte), or CD3^POS^ (T lymphocyte), populations in either gonadal or subcutaneous fat depots **(** **Figure 5B**). Neutrophils (Siglec F^POS^) were significantly reduced in both gonadal and subcutaneous depots of Ablated mice, while granulocytes (Gr-1^POS^) were significantly elevated in the gonadal fat of Ablated animals. Finally, diphtheria toxin elicits cell death through rapid inhibition of protein translation *via* ADP ribosylation of elongation factor 2 (17). Whether adipocyte death by this mechanism promotes macrophage recruitment to and engulfment of dying adipocytes was assessed by immunohistochemical staining of adipose tissue sections from Competent and Ablated mice with antibodies to the macrophage marker, CD68. Macrophage-enshrouded adipocytes (crown-like structures) were essentially absent in sections of gonadal and subcutaneous adipose tissue from both cohorts **(** **Supplementary Figure 7** **)**.

Because myeloablation could also modulate the recruitment of inflammatory cells to adipose tissue, we compared the abundance of pan-hematopoietic and myeloid cells in the gonadal fat of non-irradiated and Competent mice **(** **Figure 5B** **)**. There was no difference in the levels of these cells between the groups, suggesting that post-irradiation recruitment of inflammatory cells was minimal or had completely resolved by study end.

### HSCDA Depletion Did Not Affect Energy Balance but Was Associated With Diminished Physical Activity

**Figure 6:**
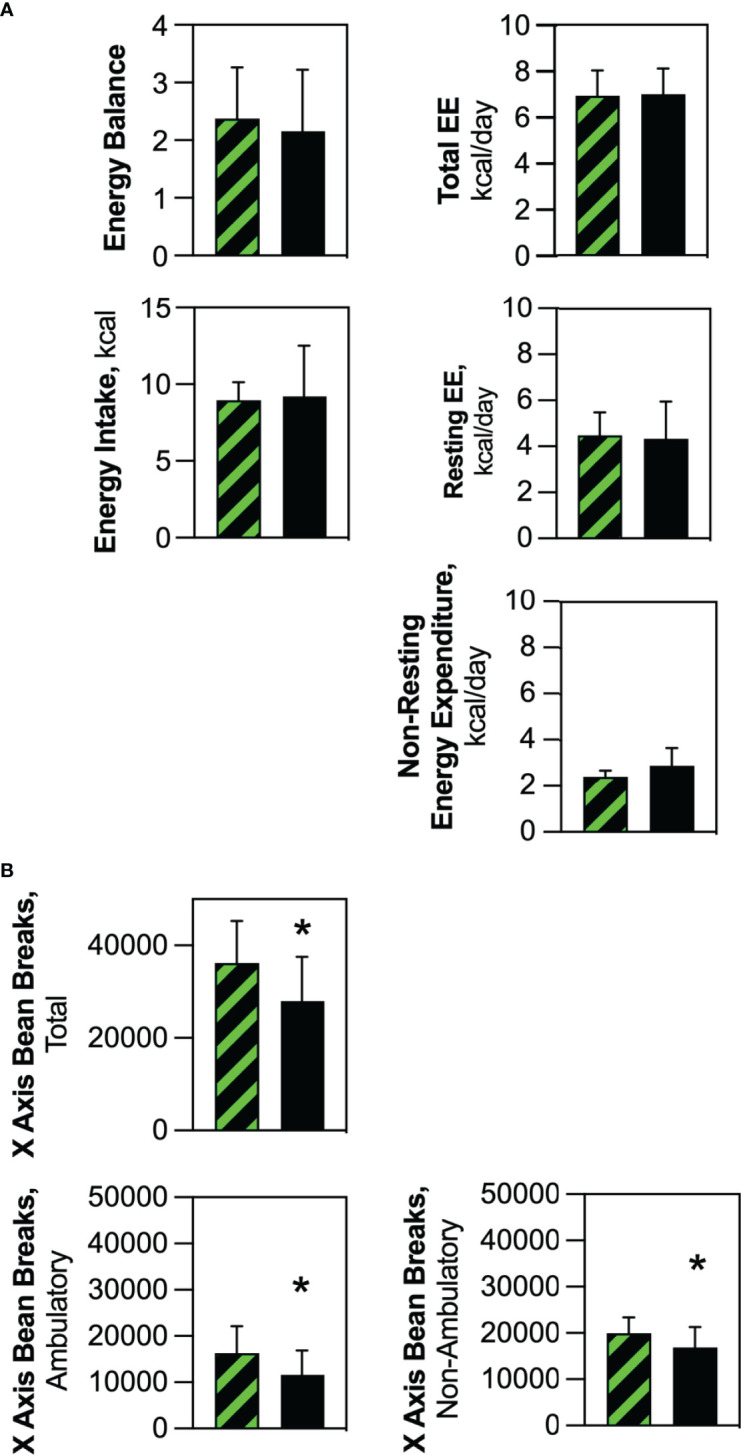
Energy balance, energy intake and energy expenditure were the same in Competent and Ablated mice, but physical activity was significantly reduced in the Ablated cohort. Individual Competent and Ablated mice were acclimated to calorimetry chambers for 72 hours, after which calorimetric endpoints and physical activity were measured for a period of 96 hours. **(A)** Energy balance, energy intake and components of energy expenditure (total, resting and non-resting) were averaged over the 96-hour measurement period for Competent (black/green crosshatch bars) and Ablated (solid black bars) cohorts. **(B)** Components of physical activity (total, ambulatory and non-ambulatory) were measured and averaged over the 96-hour measurement period for Competent (black/green crosshatch bars) and Ablated (solid black bars) cohorts. (n=8 for both cohorts, * indicates p≤0.05 B).

Indirect calorimetry showed no difference in energy intake or energy expenditure (Total, Resting and Non-Resting), or calculated energy balance (25) between Competent and Ablated cohorts when values were averaged over several days **(** **Figure 6A** **)**. However, when the data was assessed daily, we noted that energy balance and energy intake were significantly reduced every fourth day, but otherwise stable on the remaining three days **(** **Supplementary Figure 8** **)**. This pattern was consistent with data reported by Giles et al. (25) linking similar changes in these parameters to the murine estrus cycle. Furthermore, both energy balance and energy intake were constant in cohorts of transplanted ovariectomized mice, which supports the contention that the changes observed in surgery-naive Competent and Ablated mice were related to intact ovarian function. Although energy expenditure was unchanged, physical activity was significantly diminished in the Ablated mice **(** **Figure 6B** **)**. Both ambulatory and non-ambulatory physical activity were diminished in the Ablated mice.

### HSCDA Depletion Elicits Insulin Resistance Without Changes in Circulating Glucose, Non-Esterified Fatty Acids or Triacylglycerols, or Glucose Clearance

**Figure 7:**
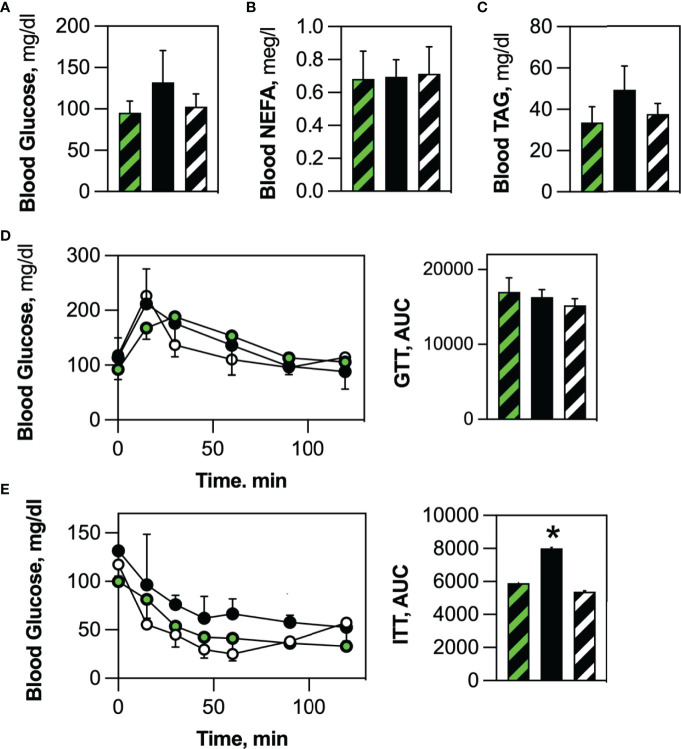
HSCDA ablation elicited insulin resistance. No differences were noted in **(A)** circulating glucose, **(B)** non-esterified fatty acid (NEFA) or **(C)** triacylglycerol serum levels between fasted transplant-naïve (black/white crosshatch bars), Competent (black/green crosshatch bars) and Ablated (solid black bars) cohorts. **(D)** Likewise, all cohorts (transplant-naïve = black/white circles, Competent=black green circles, Ablated=solid black circles) exhibited the same time course of blood glucose disposal when challenged with an oral glucose bolus. **(E)** Insulin-stimulated glucose disposal was significantly inhibited in Ablated mice. (n=8 per cohort, *indicates p≤0.05) (AUC = Area Under the Curve).

There was no significant difference in circulating glucose **(** **Figure 7A** **)**, non-esterified fatty acids **(** **Figure 7B** **)** and triacylglycerol **(** **Figure 7C** **)** levels between Competent and Ablated mice. Likewise, oral glucose tolerance tests revealed no difference in glucose clearance between the two cohorts **(** **Figure 7D** **)**. Insulin-stimulated glucose uptake in fasted mice was lower in the Ablated cohort than in Competent animals **(** **Figure 7E** **)**. When the data was expressed as Area Under the Curve, the decrease in insulin-stimulated glucose uptake was statistically significant.

## Discussion

Here we report the selective depletion of HSCDAs in mouse adipose depots and its impact on adipose tissue composition, structure, and body-wide physiological endpoints. Specific depletion of HSCDAs, but not conventional adipocytes, was achieved through adoptive transfer of donor BM HSCs from mice in which expression of a diphtheria toxin subunit A gene was controlled by the adipocyte-restricted adiponectin gene promoter. Only adipocytes arising from transplanted HSCs were ablated while adipocytes produced from tissue resident mesenchymal progenitors were spared. Thus, by leveraging the distinct developmental lineage of HSCDAs it was possible to specifically block their production and test their contributions to adipose tissue biology. Furthermore, this model is the first to inhibit a specific white adipocyte lineage while maintaining the function of the remaining adipose tissue populations.

Because HSCDAs make only a modest contribution to the total population of adipocytes throughout the body (14), it was unclear whether their ablation would significantly affect physiological endpoints attributed to the complete or partial control of adipose tissue. This concern was amplified by the incomplete ablation of HSCDAs, although their abundance was significantly reduced in the Ablated cohort. We believe that the small percentage of non-ablated HSCDAs likely represent developing fat cells in which adiponectin gene promoter activity, and therefore DTA expression, was only recently initiated.

It was surprising to observe significant changes in leptin production, physical activity and insulin sensitivity in HSCDA-depleted mice given the modest contribution HSCDAs make to the total adipocyte population **(** **Table 1** **)**. That these changes occurred while adiposity, body composition and circulating inflammatory cytokine production remained unchanged highlights the significant and specific role of HSCDAs and their distinctive phenotype among other adipocyte subpopulations.

**Table 1:** Morphological, Physiological and Inflammatory Changes Elicited by Blockade of HSCDA Production.

Changed	Unchanged
↑Adipocytes 30-40 um	Body Weight
↓Adipocytes 60-80 um	Gonadal & SubQ Depot Weight
↑Circulating Leptin, Adiponectin	%Fat, %Lean, %H_2_O
↓Physical Activity	Circulating Adiponectin, Insulin, IL-6, MCP1, tPAI-1, Resistin
↓Insulin Responsiveness (ITT)	CD45^POS^, CD11b^POS^, B220^POS^ and CD3^POS^ Cells in Adipose Stroma
↑HSCDA Progenitors (Gonadal)	Crown Structures/Dying Adipocytes
↑Conventional Adipocyte Progenitors (Gonadal)	HSCDA Progenitors (SubQ)
	Conventional Adipocyte Progenitors (SubQ)
	Energy Balance, Energy Intake or Energy Expenditure
	Glucose Uptake (OGTT)
	Blood Glucose, NEFA & TAG

HSCDA, hematopoietic stem cell-derived adipocyte(s); IL-6, interleukin-6; tPAI-1, tissue plasminogen activator-1; OGTT, oral glucose tolerance test; TAG, triacylglycerol; SubQ, subcutaneous; MCP-1, monocyte chemoattractant protein-1; ITT, insulin tolerance test; NEFA, non-esterified fatty acid. ↑, increased; ↓, decreased.

### HSCDA Depletion May Elicit Production of Conventional Adipocytes to Maintain Adipose Tissue Cellularity

Blocking the production of new HSCDAs did not affect body weight, adiposity, adipose depot weights or other measures of body composition. This may be due in part to normal production of HSCDAs in both cohorts prior to the adoptive transfer procedure. Because HSCDA ablation was restricted to those fat cells generated from the transplanted bone marrow HSCs, HSCDAs produced prior to transplant were spared. However, previous analysis of HSCDA abundance with either adoptive transfer or nonmyeloablative models has indicated that their turnover (production versus loss) is fairly constant or increases slowly over time (10, 12, 26). Thus, HSCDAs may be completely ablated as pre-transplant HSCDAs are turned-over or die, and production of new HSCDAs is blocked. It also remains to be determined whether HSCDA turnover rates are comparable to conventional-lineage adipocyte turnover.

The loss of HSCDAs post-transplant in the Ablated cohort may also elicit their replacement with new conventional adipocytes. This idea is supported by the adipocyte sizing analysis which revealed an increase in the number of small adipocytes (30-40 µm) while the percentage of larger adipocytes (60-80 µm) was reduced. If this was simply due to lipolysis and shrinkage of adipocytes that were previously larger, then one would have anticipated a decrease in depot size and adiposity, which was not observed. Moreover, conventional adipocyte progenitors (Lin^NEG^/CD29^POS^/PDGFR^POS^ stromal cells) were more abundant in Ablated versus Competent mice, perhaps as a compensatory mechanism to maintain adipose depot cellularity during HSCDA depletion.

HSCDA were also significantly more abundant in the gonadal fat of Ablated mice (9, 10). We have \accumulation is stimulated by high fat feeding and thiazolidinediones (9), and suppression of estrogen signaling in female mice (26). We surmise that expansion of the HSCDA progenitor population in the Ablated animals may reflect an attempt to normalize HSCDA production and adipose tissue cellularity.

### Myeloablation and HSC Reconstitution Had Minimal Impact on Inflammatory Endpoints

A major concern in these studies was the reliance on adoptive transfer to ablate endogenous HSCDA progenitors (HSCs and myeloid intermediates) in the recipient mice so they could be replaced with GFP^POS^ HSCs. Differentiation of reporter-labeled HSCs to HSCDAs allowed us to confirm the production of HSCDAs and quantify their abundance. We previously detected HSCDAs using several transplant and nonmyeloablative models (14). However, these experiments required adoptive transfer to limit ablation solely to HSCDAs while sparing conventional adipocytes.

Myeloablation by lethal irradiation engenders several problems (27). It effectively kills reproducing cells thus primarily targeting the highly reproductive cells of the hematopoietic/immune system. Survival of irradiated mice can be achieved by HSC/BM transplant which rescues hematopoiesis, including red cell production within six weeks. Although post-transplant survival approaches 100%, low-level body-wide inflammation is often observed for at least several months. We found that expression of three inflammatory cytokines, IL-6, resistin and tPAI-1 were all elevated in irradiated AdipoQ recipient mice compared to non-irradiated control mice. However, this increase in cytokine production was not associated with changes in the abundance of CD45^POS^ pan-hematopoietic cells, nor CD11b^POS^ myeloid cells in the adipose tissue of irradiated mice. Nor did we observe increased macrophage recruitment or crown-like structures in the adipose tissue of myeloablated, Competent or Ablated cohorts, a hallmark of elevated adipose tissue inflammation and apoptotic (28, 29) or necrotic (30) adipocyte death. Because HSCDAs produced prior to HSC transplant are spared diphtheria toxin-induced cell death, only HSCDAs produced post-transplant are ablated. The rate of DTA-induced HSCDA death may have been too low to elicit a detectable immune response.

We previously reported that the expression of several inflammatory cytokine genes was higher in HSCDAs than conventional adipocytes (10). However, our current analysis of adipose-tissue-related cytokines showed no significant differences in circulating levels of several adipokines, even when the analysis excluded the transplant-naïve cohort. This may be due to the myeloablation-induced proinflammatory environment in both Competent and Ablated cohorts. Measurement of cytokine levels at the whole-organism level may also mask changes in cytokine levels in individual cell populations and/or tissues. Circulating cytokine levels obviously reflect cytokine production not just by adipocytes, but by other cell populations throughout the body. Likewise, circulating adipokine levels often do not reflect adipokine gene expression levels (31). Thus, we currently conclude that HSCDAs are not the pro-inflammatory agents we previously proposed based on cytokine gene expression analysis alone.

### HSCDA Depletion Results in Elevated Leptin Expression, but Reduced Physical Activity

Our previous analysis of cytokine gene expression also revealed that HSCDAs produce no or low levels of the adipokine, leptin (10). Here we show that depletion of HSCDAs leads to a significant increase in circulating leptin. This supports a model in which dying HSCDAs are replaced by leptin-producing conventional adipocytes. However, leptin production in the Ablated cohort far exceeded (~245% increase over Competent cohort levels) the level anticipated by the replacement of HSCDAs (~10-20% of total adipocytes) with conventional adipocytes. Thus, leptin production by conventional adipocytes or other non-adipose sources must have been increased, or its transport across the blood-brain barrier was suppressed as observed in common models of leptin-resistance.

Leptin is recognized for its role in the maintenance of body weight and composition by promoting satiety and increasing energy expenditure. However, these effects are most notable under conditions of excess calorie intake, but are less evident under calorie-restricted, low calorie or eucaloric conditions. In our experiments the mice were fed a eucaloric, low fat/low sucrose diet. Thus, the elevated leptin levels in the Ablated cohort did not correlate with increased or reduced energy intake, increased energy expenditure or overall changes in energy balance. However, physical activity was significantly reduced in the Ablated animals. The diminished physical activity in this cohort was not accompanied by changes in body weight, adiposity or other measures of body composition. Changes in caloric expenditure due to physical activity are frequently masked by decreases in other components of energy expenditure (32).

Among agents that stimulate leptin production, no changes were observed in circulating insulin or IL-6 or circulating glucose or non-esterified fatty acids. All animals were female and none were lost due to infection, removing both estrogen (33) and bacterial lipopolysaccharide (34) as potential causes of increased leptin expression. The reduction in physical activity in the Ablated cohort, in spite of elevated circulating leptin levels, suggests that HSCDA ablation perhaps induces leptin resistance. This conclusion is not supported by the absence of body weight, adiposity, energy intake and energy expenditure differences between Ablated and Competent cohorts. However, it is worth noting that the impact of leptin on body composition and energy metabolism are most notable in models of high calorie feeding and obesity, and less so in low calorie or calorie-restricted models (35). Thus, calorie excess models may be required to reveal the full impact of HSCDAs on energy metabolism.

### HSCDA Depletion Elicits Insulin Resistance

Mice in which HSCDAs were ablated also exhibited mild, but significant decline in insulin -stimulated glucose uptake. Unstimulated glucose uptake, and fasting blood glucose and insulin levels were the same in both Competent and Ablated cohorts suggesting that the decrease in insulin-stimulated glucose uptake was due to inhibition of liver and skeletal muscle insulin sensitivity rather than diminished pancreatic insulin production. The ability of peripheral tissues to respond to insulin can be suppressed by circulating free fatty acids (36, 37) and/or inflammatory agents like IL6. TNFα and MCP-1 (38–41), but these parameters did not differ between our cohorts. Moreover, adiponectin, an adipokine that improves insulin sensitivity and attenuates inflammation was elevated in Ablated mice. Hyperleptinemia in the absence of high fat diet and/or obesity improves insulin sensitivity and glucose uptake (42, 43) and only elicits leptin- and insulin-resistance in the context of diet-induced obesity (44). Thus, while hyperleptinemia and hyperinsulinemia are frequently implicated in metabolic problems of obesity, their appearance together in our non-obese/non-high calorie diet model remains puzzling.

## Summary

Herein, we describe the impact of HSCDA depletion in mice on adipose tissue cellularity, and metabolic, physiologic and inflammatory endpoints. Previous reports from other investigators have focused on selective depletion models in which the total adipocyte population or just brown/beige adipocytes were targeted. These are the first experiments in which the consequences of blocking the production of HSCDAs, a white adipocyte subpopulation, but not other white or brown/beige adipocytes were examined. Not surprisingly, diminished production of HSCDAs, which are less abundant than conventional white adipocytes, had minimal impact on many common endpoints of adipocyte abundance and function **(** **Table 1** **)**. The minimal changes in the phenotype of HSCDA-depleted mice may also be due to our use of a conventional diet, as more profound changes in adipocyte function are typically only apparent under more extreme stimuli such as high-fat feeding, calorie restriction, cold exposure, changes in sex hormone production, etc. However, these initial results also suggest that HSCDAs play a role in the maintenance of adipose tissue cellularity as the blockade of HSCDA production did not reduce adipose depot weights, but increased the abundance of small adipocytes and conventional adipocyte progenitors. Equally important, we observed significant increases in circulating leptin levels, accompanied by diminished physical activity and insulin responsiveness. This combination of features is a hallmark of leptin resistance, and calls for additional studies to understand how the loss of HSCDAs can elicit leptin resistance without overt increases in adiposity.

## Methods

### Animal Care

Animal Studies were approved by the Institutional Animal Care and Use Committee at the University of Colorado Anschutz Medical Campus. AdipoQ cre [Jax #028020, B6.FVB-Tg(Adipoq-cre)1Evdr/J], ROSA^mT/mG^ [Jax #007676, B6.129(Cg)-*Gt(ROSA)26Sor^tm4(ACTB-tdTomato,EGFP)Luo^/J*] and ROSA^DTA^ [Jax #009669, B6.129P2-*Gt(ROSA)26Sor^tm1(DTA)Lky^/J*] and wild-type C57BL6/6 (Jax 000664, C57BL/6J) were purchased from The Jackson Laboratory (Bar Harbor, ME). Mice were group housed in polycarbonate cages in the Center for Human Nutrition Satellite Animal Facility (12:12 hr light:dark cycle, thermoneutral at 27°C) with free access to water. They were fed ad libitum a chow a low-fat diet free of sucrose and phytoestrogens (Envigo Teklad global soy protein-free extruded diet #2920X).

### HSCDA Ablation Model

Ablation of adipocytes was achieved by crossing AdipoQcre mice with animals in which Cre recombinase expression was directed by the adiponectin promoter (AdipoQcre) with mice in which a diphtheria toxin subunit A transgene was placed downstream of a floxed stop codon in the ROSA26 locus (ROSA^DTA^). Cre-mediated excision of the stop codon initiated DTA expression and cell death, which was restricted to mature adipocytes. All donor mice were crossed with flox-stop-flox mTmG reporter transgenic mice (ROSA^mTmG^) resulting in either triple transgenic mice (AdipoQcre-ROSA^mTmG^/^DTA^, designated AdipoQ DTA) or double transgenic mice (AdipoQcre-ROSA^mTmG^/^+^, designated AdipoQ) to be used as bone marrow donor animals. The use of these animals as donor mice allow us to reconstitute wild-type animals in which the production (or lack thereof) of adipocytes from bone marrow stem cells could be quantified by flow cytometry or fluorescence microscopy. Control mice carried the AdipoQ cre allele and a ROSA^mTmG^ reporter gene as this combination was necessary rather than cre-negative control mice, because Cre recombinase was required for GFP expression and adipocyte tracking.

Selective ablation of HSCDAs was achieved by transplanting HSCs from either female AdipoQ or Adipoq DTA mice into myeloablated female wild type C57BL/6. HSCs were purified from BM recovered from the femurs and tibia of euthanized donor mice. Bone marrow cells were collected from the donor animals by dislocation and removal of the hind leg. The lower leg and tissue were dissected from the femurs and the epiphyses cut off. Using a 27 gauge needle the marrow was flushed with 5ml cold HBSS + 2% FBS into a collection tube then filtered through a 100µm cell strainer and washed in HBSS, 2% FBS media.

Fluorescent antibodies to the cell surface markers Lin, Sca-1 and c-Kit were added to the resuspended bone marrow cells at 0.25 micrograms/10^6^ cells. Samples were incubated at 37°C in the dark for 25 minutes. Following incubation samples were centrifuged to remove unbound antibodies, and the cells were resuspended in PBS containing 5% FBS. Bone marrow cells were sorted within 15 minutes using a Moflo XDP cell sorter with Summit 4.3 software. The sheath fluid was Isoflow. The sample and collection tubes were maintained at 5°C. The sample flow rate was set to a pressure differential of less than 0.4 psi. Sort mode was set to Purify 1. Appropriate signal compensation was set using single color control samples. Lin^NEG^/Sca-1^POS^/cKit^POS^ cells were isolated as the murine HSC fraction for subsequent transplant into recipient mice.

Recipient mice were irradiated with two split doses separated by 4 hours of 6 Gy per dose using an X-ray source. Immediately following the second irradiation dose, mice were injected *via* the retroorbital venous plexus with 1 x 10^6^ isolated bone marrow cells from AdipoQ or AdipoQ DTA donor mice. Wild-type mice transplanted with HSC from AdipoQ donors were designated “Competent”, and mice transplanted with HSC from Adipoq DTA donors were designated “Ablated”. Engraftment was evaluated approximately 4 weeks post-transplant by flow cytometry of PBMC for membrane-localized Tomato expression from the ROSA^mTmG^ reporter present in both Competent and Ablated cohorts, and routinely approached 95% of circulating cells.

### Fractionation of Adipose Tissue and Preparation of PBMCs

Adipocyte and stromal fractions were prepared by collagenase digestion and flotation/differential centrifugation as previously described (9). PBMCs were isolated from defibrinated blood by centrifugation over Ficoll-Paque medium. Approximately 3 ml of blood were mixed with an equal volume of PBS and gently layered over 3 ml of Ficoll-Paque. The tubes were centrifuged at 500 x g for 30 minutes and the supernatant carefully removed. The PBMCs layered on top of the Ficoll-Paque were recovered, washed once with PBS and resuspended in PBS or culture medium as necessary.

### Flow Cytometry

#### Staining of Adipocytes with DAPI and LipidTOX Deep Red

Buoyant adipocytes resulting from collagenase digestion and flotation/differential centrifugation were transferred to a clean tube and in HBSS supplemented with 2% FBS and 200 nM adenosine (adipocyte wash buffer or AWB) at room temperature and gently spun to remove any remaining stromal contaminants. Adipocytes were then transferred by large bore pipette tips to a clean tube with fresh AWB added with gentle mixing. After the adipocytes accumulated at the top of the liquid, they were transferred to another fresh tube and washed with AWB an additional time.

The cells were then incubated in a 1:200 dilution of LipidTox DR for 20 minutes at room temperature. DAPI was added to a final concentration of 3.75 ng/ml and incubation continued for another 10 minutes. The cells were washed and then examined by fluorescence microscopy or flow cytometry.

#### Conventional Flow Cytometry and Sorting

Adipose stroma, PBMC or adipocytes were stained with antibodies to the cell surface markers indicated in the figures and/or legends. Gating strategies included exclusion of red blood cells with Ter199, and doublet discrimination. Controls included unstained cells and cell suspensions incubated with APC- or PE-conjugated isotype matched control antibodies. Cells were analyzed or sorted using a Legacy Moflo cell sorter with Summit 4.3 software.

### Glucose and Insulin Tolerance Tests

#### Oral Glucose Tolerance Test

Mice were fasted 3 to 4 hours prior to and for the entire duration of the study. Immediately prior to the first timepoint, mice were restrained in a rodent restrainer. The distal portion of the tail was cleaned with 70% ethanol and 1 mm or less of the tail tip was amputated. This amputation allowed sampling of blood from the tail. An initial basal blood glucose measurement was collected. The mouse was then administered a dextrose load at 2 g/kg *via* oral gavage using a sterile flexible gavage needle (18 g, 2 inch length, 2mm ball end, flexible PTFE gavage needle) and 1cc syringe. Gavage volume was kept below 1% of the animal’s bodyweight (v/v), usually less than 0.3 cc for a 30 g mouse. The mouse was then returned to normal housing conditions. At the following timepoints; 15, 30, 60, 90, and 120 minutes post-dextrose bolus, the animal tail is held, the scab removed, and the blood droplet is then sampled for blood glucose. At the conclusion of the study animals were returned to normal housing conditions.

#### Insulin Tolerance Test

Mice were fasted 3 to 4 hours prior to and for the entire duration of the study. Immediately prior to the first timepoint (T0), mice were restrained in a rodent restrainer. The distal portion of the tail was cleaned with 70% ethanol and 1 mm or less of tail tip was amputated. This amputation allowed sampling of blood from the tail. An initial basal blood glucose measurement was collected. The mouse was then administered an insulin bolus at a dose of 0.5 U/kg *via* IP injection and returned to normal housing conditions. At the following timepoints; 15, 30, 45, 60, 90, and 120-minutes post insulin bolus, a tail vein blood drop was sampled for blood glucose and recorded, and the animal was returned to normal housing.

### Indirect Calorimetry

For metabolic phenotyping, mice were moved and singly housed in a Comprehensive Laboratory Animal Monitoring System (CLAMS) Indirect Calorimetry System for a period of 7 days. The first 3 days were a ‘run-in’ period during which the animals acclimated to the new housing conditions as well as different water lixits and food hoppers. No measurements were analyzed during the run-in phase. The run-in period allowed for all necessary checks and calibration to be performed on the instruments. Following the 3 day ‘run-in’ period, metabolic measurements were collected for 4 consecutive days.

### Quantitative Magnetic Resonance Imaging (qMRI)

Mice were weighed for total body weight, then placed into a measurement cylinder. The mouse was guided to the end of the cylinder using a plunger with enough space for the mouse to turn around, but no more. The assembly was inserted into the qMRI instrument and body composition measurements were recorded over a period of approximately 2 minutes.

### Adipocyte Sizing

#### Adipocyte Sizing by Imaging Cytometry

Adipocytes were released from dissected adipose tissue by collagenase digestion into a single-cell, adipocyte suspension. A dilution of adipocytes was prepared and loaded into adipocyte counting slides. The slides were immediately viewed and counted on an imaging cytometer (Cellometer Vision CBA) using Cellometer Vision software (ver. 3.0.0.9) and adipocytes were counted using the adipocyte count mode under brightfield conditions. Counts were repeated until at least 250 total adipocytes had been counted. The following cell parameters were utilized: min. size 20 um, max. size 400 um, roundness 0.10, contrast enhancement 0.10, do not decluster clumps, decluster edge factor 0.5, decluster Th factor 1.0, background adjustment 1.0.

Morphometric Adipocyte Sizing: Phase contrast images were acquired of hematoxylin and eosin-stained adipose tissue sections at 4X magnification on a Nikon TE2000-U microscope using NIS-Elements Software. Unprocessed.tiff image files were then processed using the Adiposoft plug-in (ver. 1.16) in FIJI (ver. 2.1.0) using batch processing mode, excluding edge adipocytes, with minimum size set to 20 µm and maximum size to 150 µm.

### Measurement of Metabolic Endpoints and Adipokines

Approximately 0.75 ml blood samples were recovered from anesthetized mice immediately prior to euthanasia. Blood glucose levels were determined using a OneTouch Ultra Glucometer. Serum was prepared from each blood sample for subsequent analyses. Circulating insulin levels were measured by ELISA according to the manufacturer’s instructions. Blood serum non-esterified fatty acids and triglycerides were determined using standard clinical reagents. Plasma adiponectin, interleukin-6 (IL-6), tumor necrosis factor-α (TNF-α), interleukin-1β (IL-1β), tissue plasminogen activator inhibitor-1 (tPAI-1), leptin and macrophage chemoattractant protein-1(MCP-1) levels were measured by Luminex multiplex bead assay.

### CD68 Immunostaining

Five-micrometer sections of paraformaldehyde-fixed, paraffin-embedded adipose tissue were deparaffinized and rehydrated in a graded ethanol-water series. Sections were subjected to antigen retrieval in citrate buffer in a pressure cooker for 20 min. Sections were blocked with PBS containing 10% goat serum for 30 min at room temperature. Sections were then blocked with anti-mouse IgG (H+L) antibody to prevent non-specific secondary antibody binding to endogenous antibodies in the tissue. Sections were incubated overnight in PBS-10% goat serum at 4° C with a mouse anti-CD68 antibody. The sections were then washed and incubated with the indicated Alexa Fluor-conjugated goat anti-mouse secondary antibodies (1:250 dilution) for 1h at room temperature. Coverslips were affixed with ProLong Gold fluorescent mounting medium containing DAPI.

### Microscopy

Microscopy was performed on a Nikon TE2000-U inverted epifluorescent microscope with a motorized, remote focus/Z-axis stage controller. Brightfield and fluorescent images were captured to a desktop computer with either a DS-Fi2 color camera or a DS-QiMc black & white camera both managed by a DS-U3 PC-based camera control unit. Image data was acquired with NIS-Elements software.

### Statistics

Statistical analysis was performed using the Prism 9.2.0 program. Comparisons were performed using unpaired t tests with Welch’s correction. The data presented in graphs represent averages of values obtained from at least two experiments with 4 animals per group. Data were considered statistically significant with a *P* value <0.05.

## Data Availability Statement

The raw data supporting the conclusions of this article will be made available by the authors, without undue reservation.

## Ethics Statement

The animal study was reviewed and approved by Institutional Animal Care and Use Committee at the University of Colorado Anschutz Medical Campus.

## Author Contributions

KG, TS, JM, PM, WK, SM, and DK contributed to the conception and design of the study. TS, JM, and MJ performed the experiments. KG, TS, AL, JM, MJ, and DK analyzed the data. DK wrote the first draft of the manuscript. All authors read, edited and approved the submitted version.

## Funding

This material is the result of work supported with resources and the use of facilities at the Eastern Colorado VA Geriatric Research, Education and Clinical Center, and the University of Colorado Anschutz Medical Campus, Aurora, CO. This research was funded by VA MERIT Award CE I01BX005135 (to DK) and U.S. National Institutes of Health (NIH) National Institute of Diabetes and Digestive and Kidney Diseases Grants R01DK109547 (to DK) and U54 AG062319 (to DK, PM, and WK), and K01 DK109053 (to KG). This research was also supported in part by NIH National Center for Advancing Translational Sciences Colorado Clinical and Translational Science Awards Grant UL1 TR001082 and NIH National Cancer Institute Centers for Common Disease Genomics Grant P30 CA046934.

## Conflict of Interest

The authors declare that the research was conducted in the absence of any commercial or financial relationships that could be construed as a potential conflict of interest.

## Publisher’s Note

All claims expressed in this article are solely those of the authors and do not necessarily represent those of their affiliated organizations, or those of the publisher, the editors and the reviewers. Any product that may be evaluated in this article, or claim that may be made by its manufacturer, is not guaranteed or endorsed by the publisher.
