# Supplementary material for: Hematopoietic Stem Cell-Derived Adipocytes Modulate Adipose Tissue Cellularity, Leptin Production and Insulin Responsiveness in Female Mice
Source: Front Endocrinol (Lausanne). 2022 Jun 3;13:844877. doi: 10.3389/fendo.2022.844877 (PMC9203959; doi:10.3389/fendo.2022.844877)
Supplement: Supplementary file 1 [file DataSheet_1.pdf]

SUPPLEMENTARY MATERIALS & METHODS, & DATA

Hematopoietic Stem Cell-Derived Adipocytes Modulates Adipose Tissue Cellularity, Leptin Production, and Insulin Responsiveness in Mice.

Gavin, Sullivan, Maltzahn…Klemm

Supplementary Table 1: List of conventional PCR primers and sequences.

Primer Name	Sequence (3' -> 5')	Description
oIMR7318	CTC TGC TGC CTC CTG GCT TCT	mT/mG genotyping (common)
oIMR7319	CGA GGC GGA TCA CAA GCA ATA	mT/mG genotyping (WT R)
oIMR7320	TCA ATG GGC GGG GGT CGT T	mT/mG genotyping (WT F)
oIMR1084	GCG GTC TGG CAG TAA AAA CTA TC	cre transgene forward
oIMR1085	GTG AAA CAG CAT TGC TGT CAC TT	cre transgene reverse
oIMR7338	CTA GGC CAC AGA ATT GAA AGA TCT	internal positive control forward
oIMR7339	GTA GGT GGA AAT TCT AGC ATC ATC C	internal positive control reverse
13840	CCA AAG TCG CTC TGA GTT GTT ATC	DTA wild-type forward
13841	GAG CGG GAG AAA TGG ATA TG	DTA wild-type reverse
12211	CGA CCT GCA GGT CCT CG	DTA mutant forward
8824	CTC GAG TTT GTC CAA TTA TGT CAC	DTA mutant reverse

Supplementary Table 2: List of quantitative real-time PCR assays.

Gene	Vendor	Assay Identifier
Actb	Qiagen	QT01136772
Adipoq	Integrated DNA Technologies	Mm.PT.58.9719546
B2m	Integrated DNA Technologies	Mm.PT.39a.22214835
B2m	Thermo Fisher Scientific	Mm00437762_m1
EGFP	Thermo Fisher Scientific	Mr04329676_mr
Gapdh	Qiagen	QT01658692
Pdk4	Qiagen	QT00157248

Supplementary Table 3: Flow cytometry antibodies and fluorophore conjugates

Antibody	Fluorophore	Vendor	Catalog
CD11b	PE/Cy7	BioLegend	cat no. 101215
CD45	APC/Cy7	BioLegend	cat no. 103115
CD29	PE/Cy5	BioLegend	cat no. 102219
CD140a (Pdgfrα)	APC	BioLegend	cat no. 135907
Sca-1	AF700	BioLegend	cat no. 108141
B220	PE/Cy5	BioLegend	cat no. 103209
Ly6G/Ly6C (Gr-1)	PE/Cy5	BioLegend	cat no. 108409
Sca-1	BV650	BioLegend	cat no. 108143
CD170 (Siglec-F)	APC	BioLegend	cat no. 155507
cKit	PE	BD Bioscience	Cat no.553869
mouse FcX	-	BioLegend	cat no. 101319
Compensation beads	-	eBiosciences	cat no. 01-1111-42

Supplementary Table 4: List of key resources and reagents.

Reagent Type	Description	Vendor	Identifiers
Antibody-linked magnetic beads	Lineage Cell Depletion Kit, mouse	Miltenyi Biotech	Cat. No. 130-090-858
Biological molecule	Fibrinogen	Sigma Aldrich	cat no. F8630
Biological molecule	Thrombin	Sigma Aldrich	cat no. T9549
Biological molecule	Bovine plasminogen	Enzyme Research Laboratories	cat no. BPg
Biological molecule	Urokinase	Sigma Aldrich	cat no. U4010
Cell culture reagent	Dulbeco's modified eagle medium	MediaTech	cat no. 10-013-CV
Cell culture reagent	Fetal bovine serum	GeminiBio	cat no. A57H74L
Cell culture reagent	MesenCult Basal mouse media	StemCell Technologies	cat no. 05501
Cell culture reagent	Stem Cell Stimulatory Supplement	StemCell Technologies	cat no. 05502
Cell Separation Column	LD Columns	Miltenyi Biotech	cat no. 130-042-901
Chemical compound	DAPI	Thermo Fisher Scientific	cat no. D1306
Chemical compound	Paraformaldehyde	Electron Microscopy Sciences	cat. RT 15710-S
Chemical compound	HCS LipidTOX Deep Red Neutral Lipid Stain	Thermo Fisher Scientific	cat no. H34477
Chemical compound	Oil Red O	Sigma Aldrich	cat no. O0625
Commercial kit	RNeasy Mini Kit	Qiagen	cat no. 740104
Commercial kit	RED Extract-n-amp	Sigma Aldrich	cat no. R4775
Commercial Kit	Triglyceride Reagent	Pointe Scientific	cat no. T7532120
Commercial Kit	Triglyceride Standard	Pointe Scientific	cat no. T7531-STD
Commercial Kit	NEFA assay	WAKO Chemicals	cat no. NEFA-HR(2)
Consumable	Adipocyte chamber slides	Nexcelom Bioscience	cat no. CHT4-PD300-002
Consumable	Glucose test strips	McKesson	cat no. 06-R3051P-05
Genetic reagent sample (*M. musculus*)	C57BL/6J	Jackson Laboratory	stock no. 000664

Genetic reagent sample (*M. musculus*)	$ROSA^{mT/mG}$	Jackson Laboratory	stock no. 007676
Genetic reagent sample (*M. musculus*)	$ROSA^{DTA}$	Jackson Laboratory	stock no. 009669
Genetic reagent sample (*M. musculus*)	AdipoQ-cre	Jackson Laboratory	stock no. 010803
Instrument	Cell Sorter	Beckman Coulter	MoFlo XDP70
Instrument	Fluorescent Microscope	Nikon	TE2000-U
Instrument	Color Camera	Nikon	DS-Fi2
Instrument	Black & White Camera	Nikon	DS-QiMc
Instrument	Camera Control Unit	Nikon	DS-U3
Instrument	Imaging cytometer	Nexcelom Bioscience	Cellometer Vision CBA
Instrument	Quantitative magnetic resonance imager	EchoMRI	EchoMRI-900
Instrument	Blood glucometer	McKesson	True METRIX PRO
Instrument	Small animal indirect calorimeter	Columbus Instruments	Oxymax - CLAMS
Software	Prism	GraphPad	Ver. 9.2.1
Software	NIS Elements-AR	Nikon	Ver. 4.3.0
Software	Cellometer Vision	Nexcelom Bioscience	Ver. 3.0.0.9

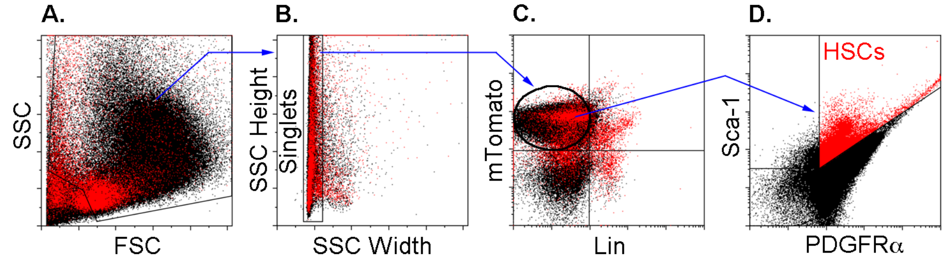

Supplementary Figure 1. Flow cytometry gating scheme for the isolation of mouse bone marrow HSCs. A) Small debris was removed from the cell population by a combination of forward scatter (FSC) and side scatter (SSC) gating. **B)** Single cells are isolated from clusters of cells by SSC Height versus SSC Width gating. **C)** Cells expressing the mTomato reporter gene but lacking mature hematopoietic Lin(eage) markers were separated from Lin^{POS} and mTomato^{NEG} populations. **D.** Lin^{NEG} cells expressing both Sca-1 and PDGFRα were isolated as murine hematopoietic stem cells (HSCs). The mTomato marker was included in the analysis to ensure that transplanted HSCs and their HSCDA progeny could be identified based on mTomato or mGFP expression, respectively.

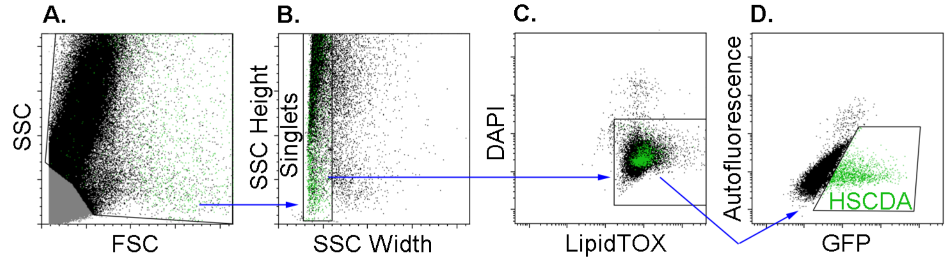

Supplementary Figure 2. Flow cytometry gating scheme for the isolation and quantitation of HSCDAs. A) Small debris was removed from the cell population by a combination of forward scatter (FSC) and side scatter (SSC) gating. **B)** Single cells are isolated from clusters of cells by SSC Height versus SSC Width gating. **C)** Live cells were identified by their lack of DAPI fluorescence, and high lipid content was confirmed by staining with LipidTOX. **D)** Finally, HSCDAs were identified by their GFP fluorescence from the autofluorescent conventional adipocyte population.

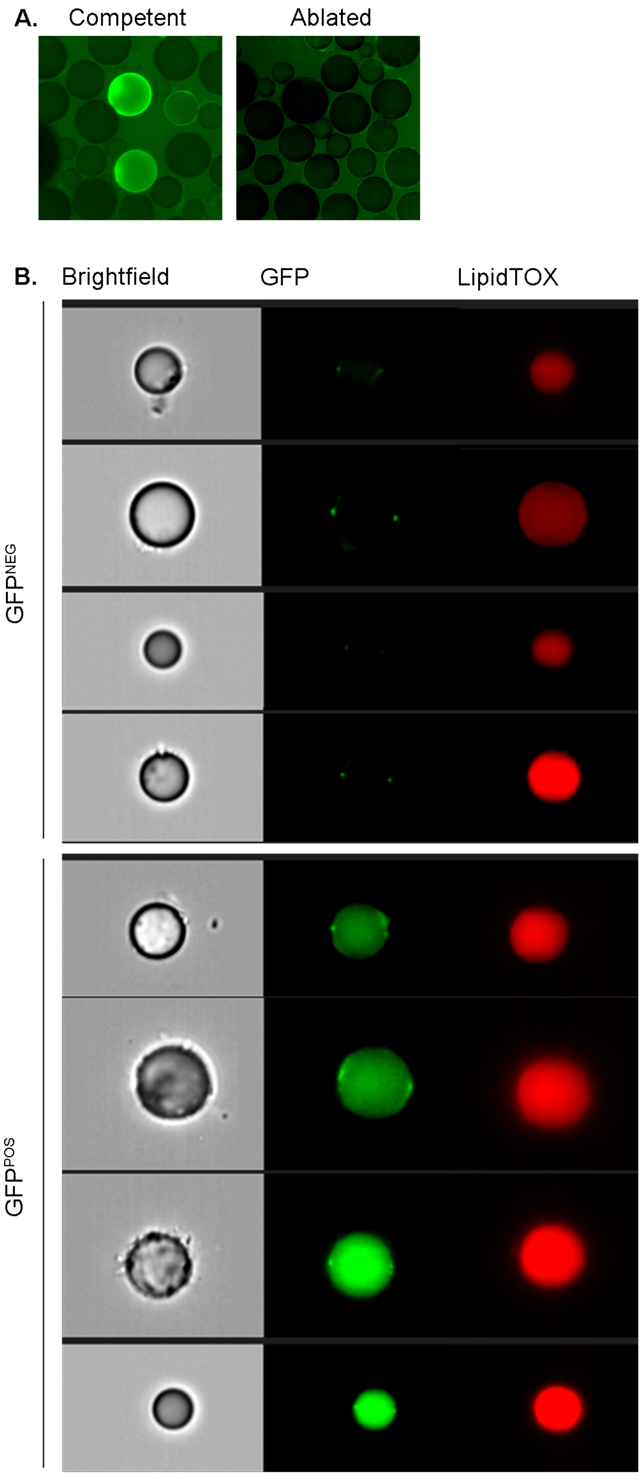

Supplementary Figure 3. GFP^{POS} HSCDAs are present in Competent mice, but not in Ablated mice. A) Representative fluorescence microscope images shows the presence of GFP-expressing adipocytes among non-fluorescent cells in free-floating adipocytes from Competent mice. GFP^{POS} adipocytes were not routinely observed in Ablated animals. **B)** Imaging flow cytometry confirmed the presence of unilocular lipid-containing ($LipidTOX^{POS}$) cells expressing GFP under the control of the adiponectin gene promoter (AdipoQ) or GFP-deficient wild type cells in Competent mice.

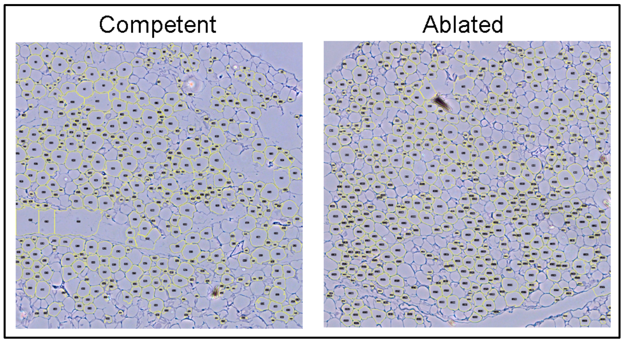

Supplementary Figure 4. AdipoSoft/FIJI images of adipocyte sizing. Representative phase contrast images of Competent and Ablated adipose tissue sections were acquired at 4X magnification Unprocessed .tiff image files were processed using the Adiposoft plug-in with FIJI to obtain adipocyte morphology.

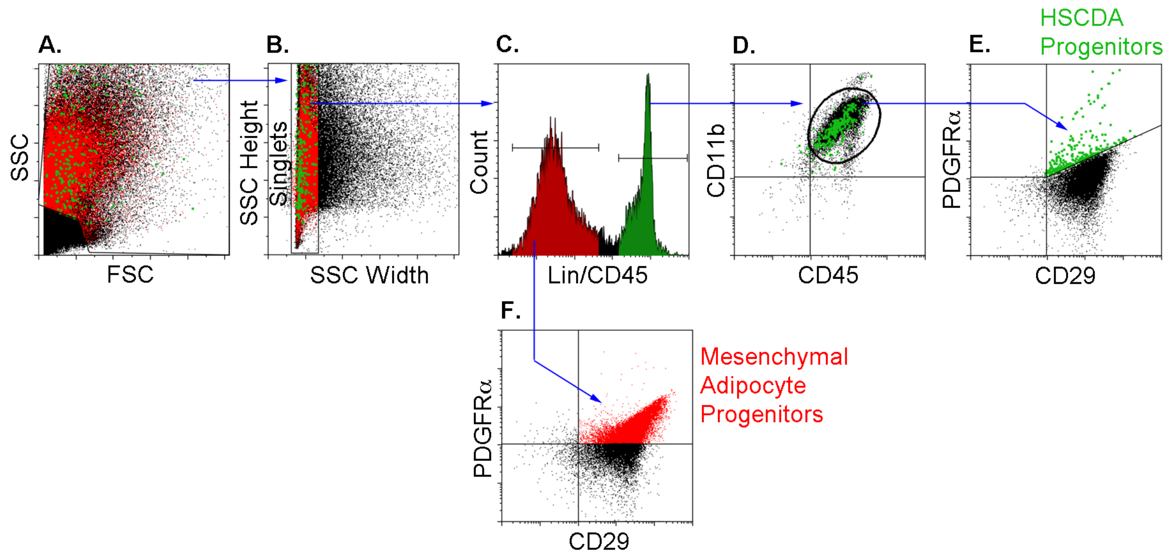

Supplementary Figure 5. Flow cytometry gating scheme for the isolation of HSCDA progenitors and conventional mesenchymal adipocyte progenitors. **A)**Small debris was removed from the cell population by a combination of forward scatter (FSC) and side scatter (SSC) gating. **B)** Single cells are isolated from clusters of cells by SSC Height versus SSC Width gating. **C)** Hematopoietic cells (green peak) expressing either Lin(eage) surface markers or CD45 were separated from mesenchymal cells (red peak). **D)** The hematopoietic population from (C) was found to consist primarily (>95%) of $CD45^{POS}$/$CD11b^{POS}$ myeloid cells. **E.** A small portion of the stromal myeloid cells expressed the mesenchymal progenitors markers CD29 and PDGFRα, and were designated HSCDA progenitors. **E)** Most of the mesenchymal cells in (C) expressed CD29, with a substantial portion also expressing PDGFRα. These cells were designated mesenchymal adipocyte progenitors.

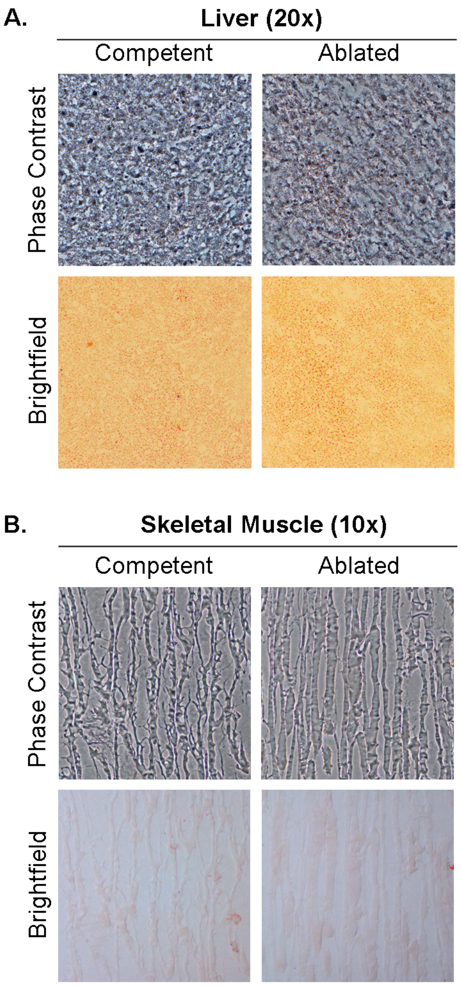

Supplementary Figure 6. HSCDA ablation does not elicit lipid deposition in liver or skeletal muscle. Liver and gastrocnemius muscle were harvested from Competent and Ablated mice and frozen in OTC. Five um sections were stained with Oil Red O solution for 10 min, briefly rinsed with 70% isopropanol then washed extensively in water. Representative Phase contrast and brightfield images are shown.

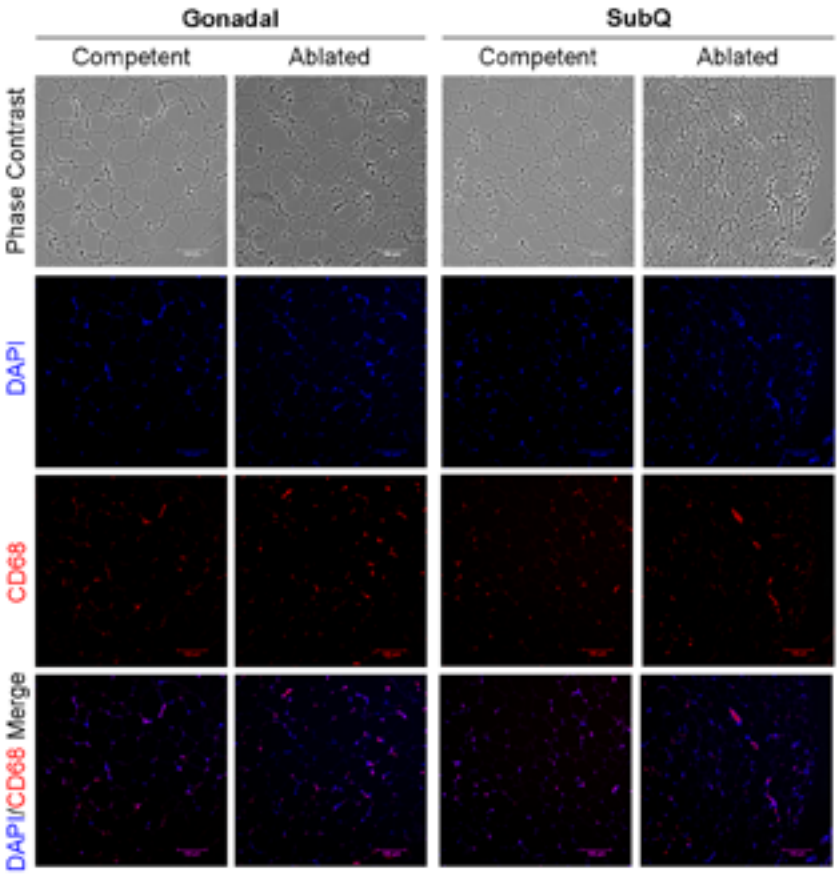

Supplementary Figure 7. Crown-like structures in the gonadal and subcutaneous (SubQ) adipose tissue of Competent and Ablated mice. Gonadal and subcutaneous adipose tissue from Competent and Abated mice was fixed, paraffin-embedded and sectioned. Deparaffinized and hydrated sections were stained overnight with antibodies to the mouse macrophage marker, CD68 and counterstained with an Alexa 555-conjugated secondary antibody. Representative phase contrast images show adipose tissue structure. Fluorescent images show DAPI-stained nuclei (blue), CD68 (red) and merged areas in magenta. All images 10x.

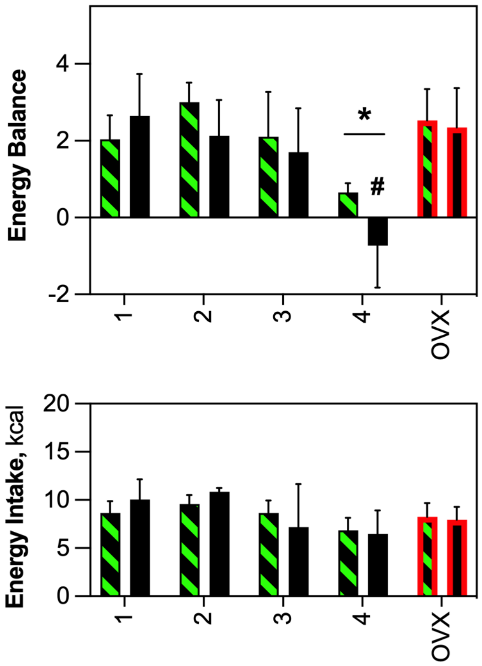

Supplementary Figure 8. Daily changes in energy balance and energy intake in Competent and Ablated mice. Surgery-naïve (thin black borders) or ovariectomized (OVX, thick red borders) Competent (black and green crosshatched) and Ablated (solid black) mice were housed individually in calorimetry chambers and acclimated for 72 hours. Energy balance and energy intake were then measured over a period of 96 hours. These parameters were relatively constant over the first 72 hours of the measurement period for surgery-naive Competent mice or for both OVX cohorts. Energy balance declined on day 4 for the surgery-naïve Competent animals.
